# Genetic structure of wild boar (*Sus scrofa*) populations from East Asia based on microsatellite loci analyses

**DOI:** 10.1186/1471-2156-15-85

**Published:** 2014-07-17

**Authors:** Sung Kyoung Choi, Ji-Eun Lee, Young-Jun Kim, Mi-Sook Min, Inna Voloshina, Alexander Myslenkov, Jang Geun Oh, Tae-Hun Kim, Nickolay Markov, Ivan Seryodkin, Naotaka Ishiguro, Li Yu, Ya-Ping Zhang, Hang Lee, Kyung Seok Kim

**Affiliations:** 1 College of Veterinary Medicine, Seoul National University, Seoul, Republic of Korea; 2 National Institute of Ecology, Seocheon-gun, Chungcheongnam-do, Republic of Korea; 3 Lazovsky State Nature Reserve, Lazo, Primorsky Krai, Russia; 4 Research Institute for Hallasan, Jeju Special Self-Governing Province, Jeju, Republic of Korea; 5 Division of Animal Genomics and Bioinformatics, National Institute of Animal Science, Rural Development Administration, Suwon, Gyeonggi-do, Republic of Korea; 6 Institute of Plant and Animal Ecology Urals Branch of Russian Academy of Sciences, Yekaterinburg, Russia; 7 Pacific Geographical Institute Far Eastern Branch of Russian Academy of Sciences, Vladivostok, Russia; 8 Laboratory of Food and Environmental Hygiene, Veterinary Medicine, Gifu University, Gifu, Japan; 9 Laboratory for Conservation and Utilization of Bio-resource and Key Laboratory for Microbial Resources of the Ministry of Education, Yunnan University, Kunming, China; 10 State Key Laboratory of Genetic Resources and Evolution, Kunming Institute of Zoology, Chinese Academy of Sciences, Kunming, China; 11 Department of Ecology, Evolution, and Organismal Biology, Iowa State University, Ames, IA, USA

**Keywords:** Microsatellites, East Asia, Genetic diversity, Genetic structure, Wild boar

## Abstract

**Background:**

Wild boar, *Sus scrofa*, is an extant wild ancestor of the domestic pig as an agro-economically important mammal. Wild boar has a worldwide distribution with its geographic origin in Southeast Asia, but genetic diversity and genetic structure of wild boar in East Asia are poorly understood. To characterize the pattern and amount of genetic variation and population structure of wild boar in East Asia, we genotyped and analyzed microsatellite loci for a total of 238 wild boar specimens from ten locations across six countries in East and Southeast Asia.

**Results:**

Our data indicated that wild boar populations in East Asia are genetically diverse and structured, showing a significant correlation of genetic distance with geographic distance and implying a low level of gene flow at a regional scale. Bayesian-based clustering analysis was indicative of seven inferred genetic clusters in which wild boars in East Asia are geographically structured. The level of genetic diversity was relatively high in wild boars from Southeast Asia, compared with those from Northeast Asia. This gradient pattern of genetic diversity is consistent with an assumed ancestral population of wild boar in Southeast Asia. Genetic evidences from a relationship tree and structure analysis suggest that wild boar in Jeju Island, South Korea have a distinct genetic background from those in mainland Korea.

**Conclusions:**

Our results reveal a diverse pattern of genetic diversity and the existence of genetic differentiation among wild boar populations inhabiting East Asia. This study highlights the potential contribution of genetic variation of wild boar to the high genetic diversity of local domestic pigs during domestication in East Asia.

## Background

Wild boar, *Sus scrofa*, is one of the most widely distributed mammalian species, native throughout Europe, North Africa, and much of Asia as far south as Indonesia. Wild boar populations have also been artificially introduced in some areas of the world including the Americas and Australasia, principally for hunting, or through escapes from captivity. *Sus scrofa* is the most common wild ancestor of the domestic pig, with which it freely hybridizes [1]. The Family Suidae includes many species of pigs, hogs and boars which served as one of the main food resources for humans during the extended history of human settlement. Their economic value increased as they were domesticated, reared, crossed, translocated, hunted, eaten, and in certain cases, venerated or persecuted for cultural or ritual purpose [2].

Since wild boar is a co-existing wild ancestor of domesticated pig, the patterns and origins of pig domestication worldwide are of increasing interest, not only in economic contexts, but also academically. Previous phylogenetic studies based on the mtDNA D-loop sequence revealed that continental wild boars and domestic pigs are clearly divided into eastern and western clades [3-5]. These studies suggested that pig domestications occurred independently in multiple centers of Eurasia, implying that European and Asian domestic populations derived from their respective regional areas. Molecular genetic evidence for the origin of wild and domestic pigs from Asia and Europe supports the historical record that Asian pigs were subsequently interbred with European breeds during the 18^th^ and 19^th^ centuries after independent domestication [6]. A recent study based on single nucleotide polymorphism (SNP) genotyping revealed that populations of wild boars from Europe and Near Eastern Asia are genetically differentiated, supporting previous mitochondrial studies [7].

It has been well known that the cosmopolitan wild boar originated and spread from Islands of Southeast Asia [3]. Knowledge of genetic diversity of wild boar in East Asia, therefore, is important for reconstructing the evolutionary history of the species as well as understanding the domestication process of local domestic pigs. Most genetic studies on wild boars in East Asia have been carried out using mtDNA sequence analysis, which did not expose geographic structure, although they revealed several subclades [4,8-10]. One recent study based on both mtDNA and nuclear genes demonstrated that no population substructure exists in either wild boars or domestic pigs in East Asia and showed a very high level of admixture between them [11]. Korean wild boars clearly clustered with Asian wild boar groups, sharing the same cluster with populations from Myanmar and Thailand [9], and the Vietnamese wild pig haplotype [8]. On the other hand, Larson *et al*. [12] ascertained that wild boars in South Korea belong to groups unique within East Asia, and remain differentiated from domestic pigs. Thus, genetic research has been conducted on domesticated pigs and wild boars in East Asia over several decades, but the patterns of genetic diversity and genetic structure of populations at a regional scale in East Asia remains unclear.

In this study, we aimed to characterize genetic relationships and genetic structure of wild boars from East Asia by examining genetic variation at microsatellite loci for a total of 238 wild boar individuals from six countries. Our results shed light on the genetic relationships among populations and help define population boundaries of wild boar in East Asia.

## Results

### Genetic characteristics and genetic diversity of wild boars in East Asia

In total, 273 alleles were observed across the 16 microsatellite loci. The number of alleles per locus ranged from ten for locus Sw72 to 33 for locus S0005, with a mean of 17.1. A total of 75 of 273 alleles were unique to single sample locations in this study. The proportion of most private alleles at a location was low, with a frequency of less than 5%, but eight of the 75 private alleles were present at a frequency over 15%: Japan (one allele of 15.6%), Yunnan, China (one allele of 20.0%), Vietnam (two alleles of 19.2% each), and Indonesia (four alleles of 14.6%, 29.2%, 45.8%, and 66.7%, respectively). The highest number of alleles (154 alleles) was found in wild boars from Indonesia, of which 33 were private alleles. Inbreeding coefficients, *F*_IS_, ranged from 0.017 to 0.279 with a mean of 0.091. Most of the populations except for two sample locations, Japan and Vietnam, showed non-significant *F*_IS_ values, implying no signature of significant inbreeding (Table 1).

**Table 1:** Genetic diversity estimates for wild boars from East Asia

**Location (Abbr.)**	**N**	**Allelic diversity (*Ad*)**	**Allelic richness (*Ar*)**	***H***_**E**_	***H***_**O**_	***F***_**IS**_
South Korea						
Gyeonggi-do (KGGW)	17	4.8	4.3	0.614	0.563	0.086^NS^
Gangwon-do (KGWW)	53	5.8	4.6	0.661	0.647	0.022^NS^
Gyeongsang-do (KGSW)	26	6.1	5.1	0.705	0.673	0.046^NS^
Jeolla-do (KJLW)	12	3.4	3.4	0.506	0.422	0.172^NS^
Jeju Island (KJIW)	37	4.0	3.1	0.549	0.539	0.019^NS^
Russia Primorsky (RUPW)	30	7.6	5.9	0.736	0.710	0.036^NS^
Japan (JPNW)	16	6.2	5.2	0.650	0.473	0.279^*^
China Yunnan (CYNW)	10	8.0	8.0	0.845	0.831	0.017^NS^
Vietnam (VIEW)	13	9.1	8.3	0.859	0.836	0.028^NS^
Indonesia (INDW)	24	9.6	7.3	0.796	0.658	0.177^*^

N: Number of samples; *Ad*: Mean number of alleles; *H*_O_: Observed heterozygosity; *H*_E_: Expected heterozygosity; *F*_IS_: Inbreeding coefficients; ^*^Significant, ^NS^Not significant, after adjusted nominal level (5%): 0.00031; *Ar*: The number of genes obtained from Yunnan, China, the smallest sample size in this study, was employed.

Levels of genetic diversity for regional samples of 238 wild boars from East Asia are shown in Table 1. The mean number of alleles across loci ranged from 3.4 (Jeolla-do, Korea) to 9.6 (Indonesia). Four diversity measures revealed a consistently high level of genetic diversity in wild boars from southeastern China (Yunnan province), Vietnam, and Indonesia (≥ 0.796 in *H*_E_ and ≥ 7.3 in allelic richness), followed by the Russian Far East (Primorsky Krai) and mainland Korea (except Jeolla-do). The lowest level of genetic diversity was found in the samples from Jeolla-do, Korea (*H*_E_ = 0.506; *Ad* = 3.4; *Ar* = 3.4), and Jeju Island (*H*_E_ = 0.549; *Ad* = 4.0; *Ar* = 3.1) (Table 1).

### Genetic relationships and gene flow among populations

Between population genetic differences, as indicated by pairwise *F*_ST_ estimates and the estimated number of migrants per generation (*Nm*), are presented in Table 2 for each pair of wild boar populations. Pairwise *F*_ST_ values ranged from 0.020 (Gyeonggi-do vs. Gangwon-do, Korea) to 0.314 (Jeolla-do vs. Jeju Island, Korea). Gene flow estimates (*Nm*) derived from *F*_ST_ ranged from 0.546 to 12.250. All wild boar population pairs were significantly differentiated from one another except population pairs from the north-central region of Korea. The wild boar population on Jeju Island showed the highest degree of genetic differentiation from other populations (mean *F*_ST_ = 0.253). The lowest mean *F*_ST_ value was found in southeastern Korea (Gyeongsang-do) vs. other populations (mean *F*_ST_ = 0.123).

**Table 2:** Genetic distances and gene flow estimates among wild boars from East Asia

	**KGGW**	**KGWW**	**KGSW**	**KJLW**	**KJIW**	**RUPW**	**JPNW**	**CYNW**	**VIEW**	**INDW**
KGGW		12.250	3.718	1.384	0.669	1.887	1.094	1.221	1.066	0.750
KGWW	0.020^NS^		6.507	2.275	0.770	2.225	1.179	1.428	1.131	0.814
KGSW	0.063^*^	0.037^*^		2.410	0.907	2.131	1.342	2.177	1.511	1.073
KJLW	0.153^*^	0.099^*^	0.094^*^		0.546	1.265	0.679	0.902	0.809	0.633
KJIW	0.272^*^	0.245^*^	0.216^*^	0.314^*^		0.770	0.640	1.170	0.734	0.673
RUPW	0.117^*^	0.101^*^	0.105^*^	0.165^*^	0.245^*^		1.170	2.154	1.575	1.179
JPNW	0.186^*^	0.175^*^	0.157^*^	0.269^*^	0.281^*^	0.176^*^		1.373	1.274	1.013
CYNW	0.170^*^	0.149^*^	0.103^*^	0.217^*^	0.176^*^	0.104^*^	0.154^*^		4.136	2.275
VIEW	0.190^*^	0.181^*^	0.142^*^	0.236^*^	0.254^*^	0.137^*^	0.164^*^	0.057^*^		2.044
INDW	0.250^*^	0.235^*^	0.189^*^	0.283^*^	0.271^*^	0.175^*^	0.198^*^	0.099^*^	0.109^*^	

Pairwise *F*_ST_ (below diagonal) and gene flow (*Nm*) estimates (above diagonal) among geographic populations of wild boars in East Asia (see Table 1 for location abbreviations).

^*^Significant after Bonferroni correction (*P* < 0.05); ^NS^Not significant; Indirect indicator of gene flow (*Nm*) was calculated among geographic populations using the equation, *Nm* =1/4{(1- *F*_ST_)/*F*_ST_}.

**Figure 1:**
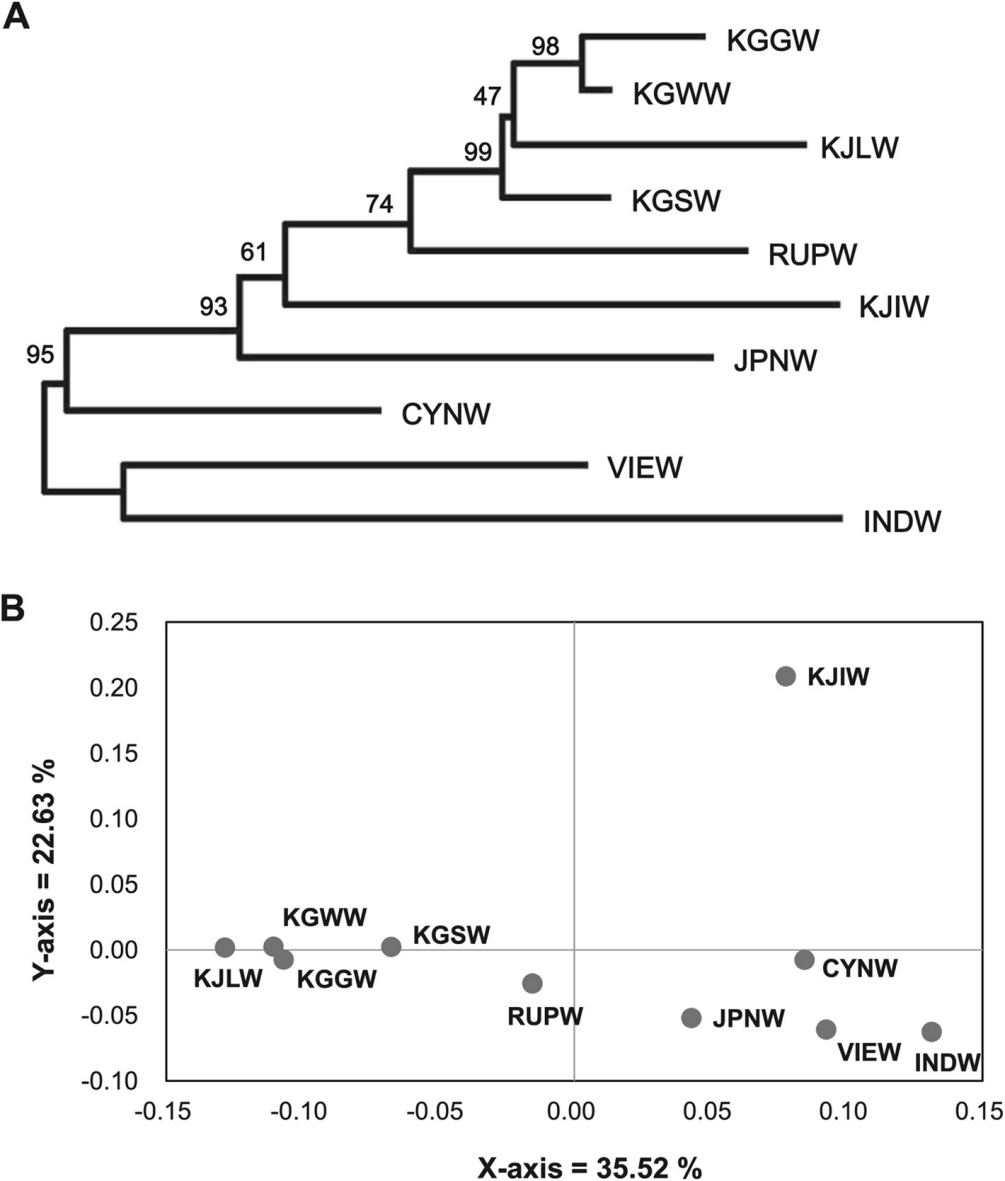
**Genetic relationships among wild boars in East Asia. A**. NJ tree based on Nei’s D_A_ distance with bootstrap values from 1,000 replications. **B**. Principal Coordinates Analysis (PCA) based on pairwise *F*_ST_’s (see Table 1 for location abbreviations).

The NJ tree based on Nei’s D_A_ genetic distance showed wild boars from Vietnam and Indonesia grouped together, forming a basal cluster to all other populations (Figure 1A). Among Korean wild boars, mainland populations grouped with, and were closely related to, wild boars from the Russian Far East (Primorsky Krai), whereas wild boars from Japan and Jeju Island were basal to Northeast Asian clades. Wild boar populations from Southeast Asia formed distinct clades from those of Northeast Asian populations.In a Principal Coordinates Analysis (PCA), the first two components, PC 1 and PC 2 (x- and y- axes, respectively), accounted for 35.52% and 22.63% of the total variance (Figure 1B). PC 1 revealed the genetic difference between wild boars by geographical isolation. “Northern” regions (mainland South Korea and Russian Far East) and “southern” regions (southeastern China, Vietnam and Indonesia) formed separate groups, with Japanese wild boars intermediate between them. The discrete position of wild boars from Jeju Island along PC 2 reflects its high genetic differentiation from all other wild boar populations.

**Figure 2:**
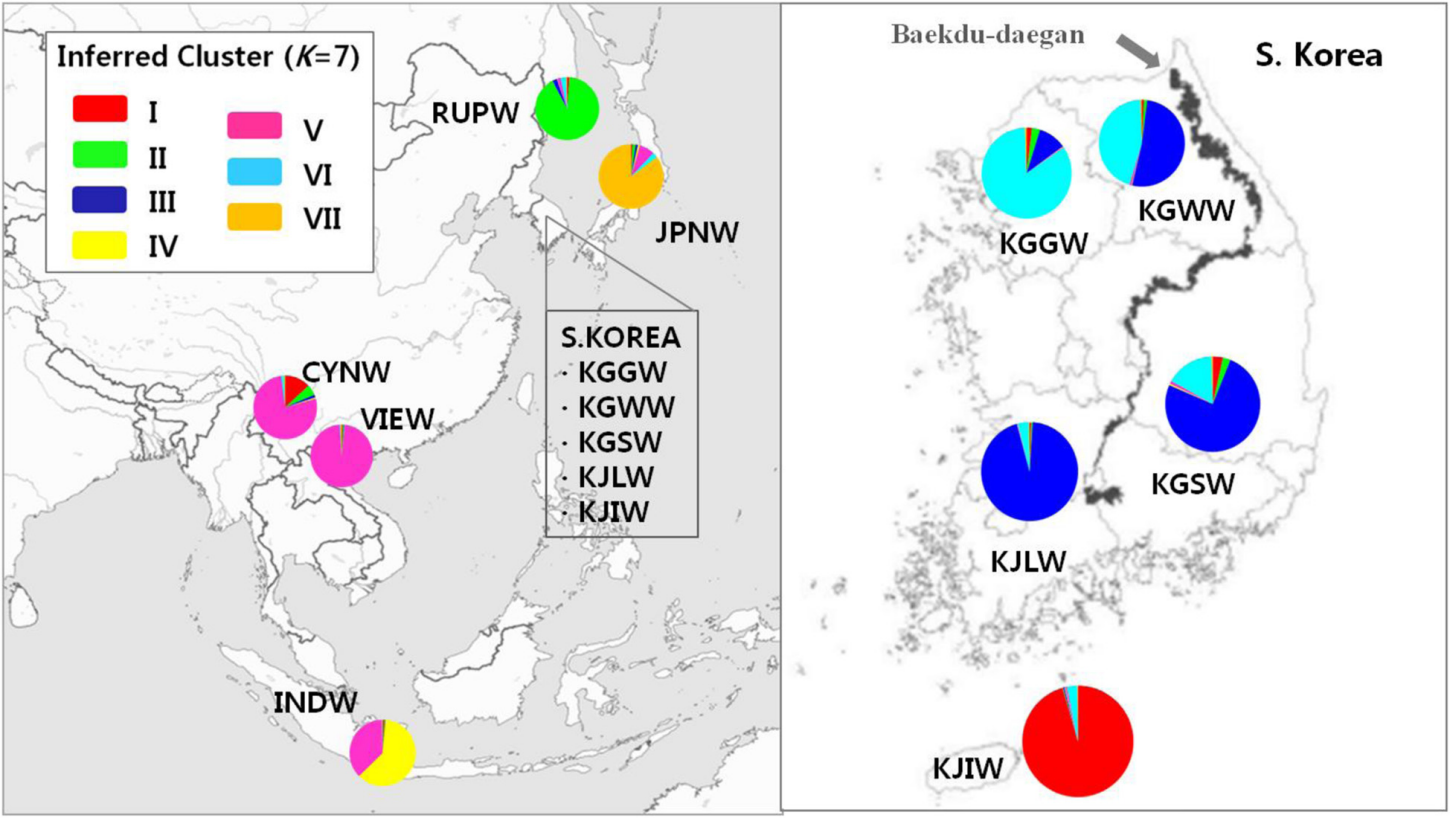
**Geographical locations of wild boar samples in East Asia (left) and South Korea (right).** Pie charts indicate proportions of membership of each sampled population to seven clusters inferred by structure analysis (*K* = 7) (see text for details). See Table 1 for location abbreviations.

**Figure 3:**
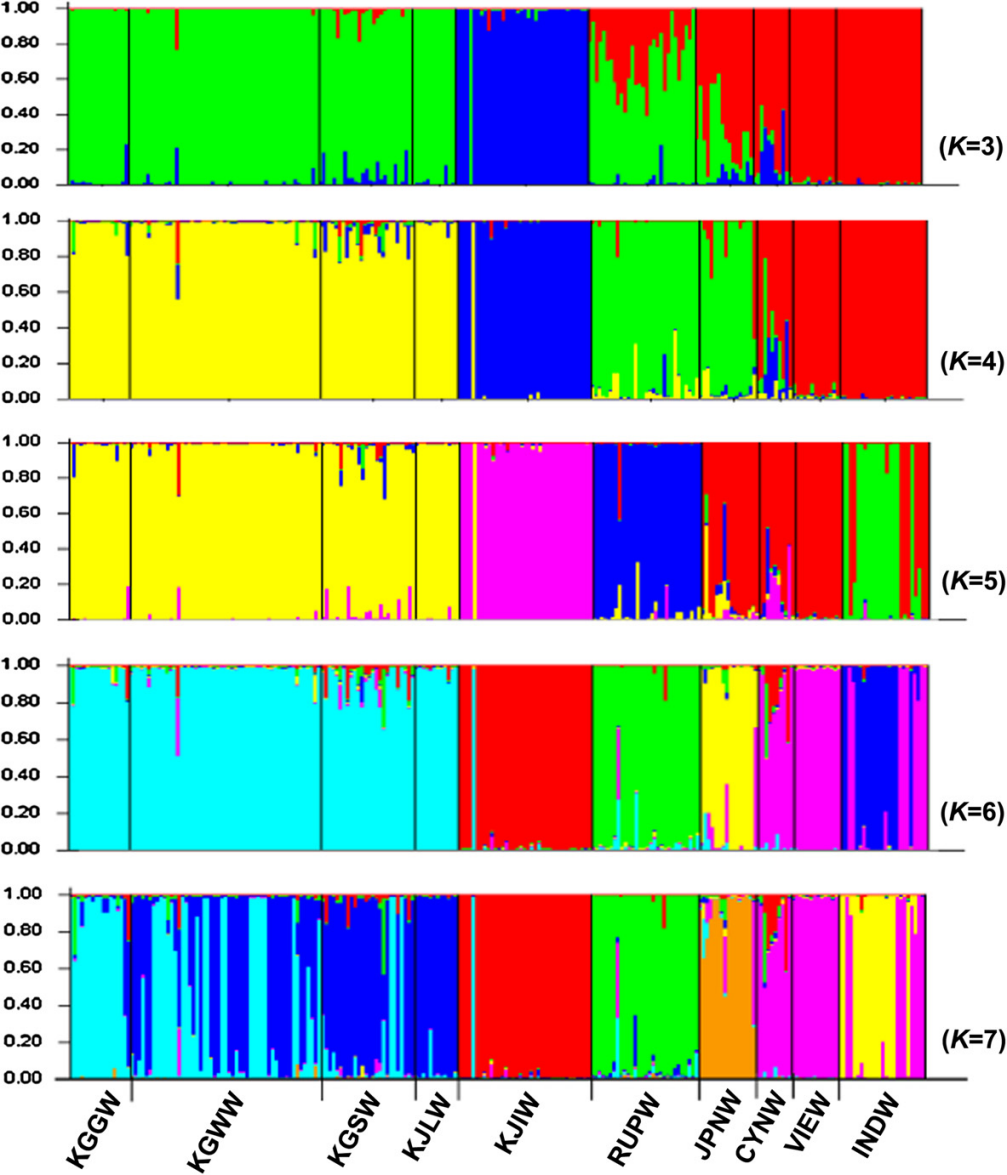
**Individual membership of wild boar samples from East Asia to the K clusters inferred by structure analysis.** Codes on the x-axis indicate the putative population of origin. See Table 1 for location abbreviations. Each color denotes a cluster from the structure analysis.

Pairwise *F*_ST_ data, the genetic relationship tree and the PCA scattergram indicate that Jeju Island wild boars are quite distinct from wild boars in mainland Korea. Interestingly, despite the genetically distinct population structure of wild boars from Jeju Island, one of the 37 individuals we sampled belonged genetically to a population from the Korean mainland (Figures 2 and 3). In addition, some wild boars on Jeju Island shared genetic profiles similar to wild boars from Yunnan province (Figure 3) and the pairwise *F*_ST_ value was relatively low.

### Genetic structure of East Asian wild boars

Samples from ten geographic locations were tested to determine the potential number of populations (*K*) they represent. Model-based clustering analysis revealed that wild boars in Eastern Asia had the highest Δ*K* when *K* was set to 3, assuming three inferred populations: 1. mainland Korea (KGGW, KGWW, KGSW, KJLW); 2. Jeju Island (KJIW); and 3. Southeast Asia (CYNW, VIEW, INDW) (Figure 3 & Additional file 1: Figure S1). In this scenario, wild boars from Primorsky Krai, Russia and Japan showed genetic compositions intermediate between mainland Korea and Southeast Asian populations. Wild boars from Primorsky Krai, Russia and Japan clustered together when *K* = 4. When *K* was set to 5, the Japanese wild boar population grouped with Yunnan province, China and Vietnam. The Indonesian population was isolated, albeit 9 of 24 individuals shared genetic composition with those populations. In the case of *K* = 6, most wild boars from Japan formed a unique genetic composition. Finally, when *K* was set to 7, the wild boars of mainland Korea were divided into two main substructures, a north-central region (KGGW and KGWW) and southern region (KGSW and KJLW), although they displayed a genetically admixed pattern (Figure 3).

When a hierarchical island model [13] was applied to verify possible substructure in each cluster, results corresponded to genetic clustering obtained when *K* was set to 7. Therefore, a total of seven genetically substructured groups of populations were found in wild boars in East Asia (Figure 3). Most wild boars from Jeju Island (KJIW), Primorsky Krai (RUPW), Japan (JPNW) and Indonesia (INDW), showed discrete genetic composition in the structure analysis, with genetic traits of the first two populations shared among a few individuals of Yunnan province, China (CYNW) (Figure 3). With one exception, wild boar individuals from Jeju Island had a conspicuously different genetic composition with respect to populations from mainland Korea. Although wild boars from mainland Korea were genetically admixed, the genetic composition showed a gradual geographic gradient from north to south (Figure 2). The structure analysis revealed that the prevalent (96%) cluster in the wild boar population on Jeju Island was more abundant (13%) in wild boars from Yunnan province than in wild boars from mainland Korea (<2%) (Figure 2).

AMOVA analysis was carried out to ascertain hierarchical patterns of genetic variation for three regions distinguished on the basis of geographical distance, pairwise *F*_ST_ and population structure (Table 3). 9.5% of genetic variance was accounted for among the three regions (*F*_RT_ = 0.095) and 11.4% among populations within region (*F*_SR_ = 0.114), to explain the proportion of genetic variance among populations to the total (*F*_ST_ = 0.198) (Table 3).

**Table 3:** Analysis of Molecular Variance (AMOVA) of wild boars from three geographic regions

**Source**	**df**	**SS**	**MS**	**Est. Var.**	**%**
Among regions	2	290.580	145.290	0.643	9%
Among populations	7	241.790	34.541	0.702	10%
Among individuals	228	1327.662	5.823	0.389	6%
Within individuals	238	1200.500	5.044	5.044	74%
Total	475	3060.532		6.778	100%
***F*-statistics**	**Value**	**P-value**			
*F*_RT_	0.095	0.001			
*F*_SR_	0.114	0.001			
*F*_ST_	0.198	0.001			
*F*_IS_	0.072	0.001			
*F*_IT_	0.256	0.001			

Three regions; North-East region (RUPW, KGGW, KGWW, KGSW, KJLW, JPNW), Jeju Island (KJIW) and South-East region (CYNW, VIEW, INDW). See Table 1 for location abbreviations.

df: Degrees of freedom; SS: Sums of squares; MS: Mean squares; Est. Var.: Estimated variance within and among populations.

The isolation by distance test revealed that genetic distance was not significantly correlated to the geographic distance for total populations (R^2^ = 0.078; *P* = 0.140). However, when the Jeju Island population was excluded, a significant regression was detected (R^2^ = 0.391; *P* = 0.002) (Additional file 1: Figure S2).

## Discussion

Levels of genetic diversity and the structuring of geographic populations provide important clues to local adaptation and species evolution. Such information can further be employed to understand the effect of genetic variation of regional wild boars on pig domestication in East Asia and to facilitate conservation and management of this species at a regional scale. In this study, wild boar populations from East Asia showed various levels of genetic diversity, as well as a distinct genetic structure, related to geographic distribution.

### Genetic diversity and population structure of wild boar in East Asia

The pattern and magnitude of allelic diversity vary with the geographic distribution of wild boars in East Asia. Wild boars from southeastern regions, represented by Yunnan province of China, Vietnam and Indonesia, exhibited generally high levels of genetic diversity with large numbers of alleles. In contrast, relatively low levels of genetic diversity were found in wild boars from Northeast Asia, except Primorsky Krai, Russia which has an intermediate level of allelic diversity.

The high level of genetic diversity and large numbers of alleles in wild boars from Southeast Asia are expected given the historical geographic range of *S. scrofa*. Previous studies [3,12,14] revealed that *S. scrofa* originated from Islands of Southeast Asia, i.e. an “ISEA” origin of wild boar. Although various factors such as climatic fluctuations and human-mediated translocations can affect the genetic composition of a spreading species, its gene pool will be retained with a higher probability in the area of origin than in areas of colonization. Additionally, extensive inter-specific gene flow in the genus *Sus* took place during glacial periods when a land bridge formed between the islands of Southeast Asia [14], and this could explain the observed high level of genetic diversity in ISEA.

Structure analysis using the hierarchical island model revealed that Indonesian wild boars are differentiated from other populations of Southeastern Asia, despite some individuals with genetic profiles similar to those of wild boars from Yunnan province and Vietnam (Figures 2 and 3). In addition, the high proportion of private alleles and high allelic diversity in the Indonesian wild boar population support its subspecific classification as the “Indonesian race”*, S. s. vittatus*, proposed by Groves and Grubb [15].

In contrast, wild boars from most of mainland Korea and Jeju Island had genetic diversity almost two fold lower than wild boars from Southeast Asia. The wild boar population from Jeju Island (*H*_E_ = 0.549; *Ar* = 3.1) exhibited the lowest genetic diversity among all populations sampled from East Asia. Negligible gene flow from the Korean mainland (mean *Nm* = 0.764, Table 2), and the sudden population increase on Jeju Island during recent decades, could account for the low level of genetic diversity on the island, and suggest there has not been enough time to reach mutation/migration-drift equilibrium since human-mediated translocation or natural migration.

Patterns of genetic diversity and differentiation at local and regional scales observed in this study, together with results from the model-based structure analysis, suggest that wild boars in Northeast Asia share closer ancestry with wild boars in southern China than do those in Vietnam and Indonesia, indicating gradual gene flow from ISEA through Southern China (Figure 2). A diverging gene pool and high level of genetic diversity in wild boars from East Asia are likely reflected in a high diversity of local pig breeds in Asia, arising during multiple and independent domestication events in this region [16].

In contrast to a previous study based on mtDNA and nuclear genes [11], which found no genetic structure among wild boar populations in East Asia, we found high genetic variation and differentiation between wild boar populations at both local and regional levels. Mitochondrial DNA sequence comparisons indicated that genetic clusters of wild boars from East Asia, including China, Korea, Japan and the Russian Far East, were not clearly separated by region [10]. In addition, no conspicuous genetic structure in East Asia, including China, Korea and Japan, was detected based on three different marker systems, mtDNA, microsatellite and Y-chromosome genes [17]. In these cases, the number of samples and markers used for wild boar study in East Asia probably were not enough to detect population structure. Alternatively, the use of populations such as domestic pigs with strong geographic structuring could mask the hidden structure of wild boars in East Asia that might otherwise exist in such region. Our contrasting results relative to previous studies [10,11,17] could also be due to the use of different marker systems. Although both mtDNA and microsatellite loci analyses showed indication of population structuring in European wild boars [18-20], microsatellite loci have shown better resolution in detecting genetic structure among geographic populations than mtDNA [18]. Population differentiation and admixture in the recent past can be better detected by fast-evolving markers like microsatellites.

Geographical distance was significantly correlated with genetic distance when the unique Jeju population was excluded (Additional file 1: Figure S2). A hierarchical genetic differentiation related to geographical distances is also well-supported by the AMOVA incorporating three regions (Table 3). Furthermore, Principal Coordinates Analysis (PCA) showed the wild boar populations in East Asia occupied unique positions along PC 1, mainly related to geographic distribution. Taken together, our data indicate that genetic differentiation of wild boars in East Asia is maintained by geographic separation.

### Genetic status of local wild boar populations in South Korea

Archaeological evidence suggests that wild boars appeared on the Korean peninsula in the mid-Pleistocene, ca. 780,000 to 130,000 years before present [21]. However, predators, such as wolf and tiger, which have played important roles in effectively controlling the population size of wild boar, have been absent from South Korea over recent decades. As a result, wild boar is the largest mammal with an extensive distribution in South Korea, although Asiatic black bears (*Ursus thibetanus*) were reintroduced to the mainland a decade ago [22]. Archaeological evidence and ancient records indicate that wild boars became established on Jeju Island, the largest island in southern Korea, presumably between the 1st and 8th centuries A.D. [23,24]. Modern populations decreased and went undetected for several decades, but over the last decade, wild boars have greatly increased on the island. Although the reason for the recent increase of wild boars on Jeju Island is unclear, it has been assumed that some captive individuals escaped to the wild. As a consequence of wild boar population growth on the mainland and Jeju Island in South Korea, proper management of the species is of increasing concern, and population genetics would be a useful tool to reveal whether gene flow occurs between local wild boar populations.

Structure analysis (*K* = 7) showed that wild boars from mainland Korea are represented by two genetic clusters (Figures 2 and 3). Although genetic traits within populations in mainland Korea were not clearly discrete, genetic profiles were gradually displaced from the north-central region (KGGW and KGWW) to the southeast region (KGSW), followed by the southwest region (KJLW) (Figure 2). Pairwise *F*_ST_ and gene flow estimates (*Nm*) support a gradual cline in genetic structure in mainland Korea (Table 2). These three regions of the Korean peninsula are geographically separated by the Baekdu-daegan mountain range, which runs most of the length of the eastern peninsula, from Baekdu Mountain in the north to Jiri Mountain in the mid-south. This mountain range may function as a geographical barrier to wild boar dispersal, although they are capable of crossing mountain ridges. Moreover, *S. scrofa* does not tend to disperse long distances from their birth site, with geographic ranges less than 6.5 Km^2^[25,26].

Our result showed that Jeju wild boar had a closer relationship with Yunnan rather than the mainland Korea, which suggests that wild boars in Jeju Island share closer common ancestry with wild boars in Yunnan, China than mainland Korea. This is in agreement with the conclusion of a previous study that Jeju Island wild boars probably introduced from somewhere in China [27], and were not directly originated from mainland Korea. A phylogenetic study using mitochondrial sequences suggested that wild boar from Jeju Island should be allocated to the Chinese wild boar cluster [27]. However, precise identification of the geographic origin of the Jeju Island wild boar will require a survey of more samples from broadly spaced regions using a variety of analytical methods, such as paternal history using Y-chromosome genes and maternal history using mitochondrial DNA.

For effective management of wild boars in Korea, genetic traits must be considered to establish appropriate strategies. Our results show that wild boar populations on mainland Korea are genetically structured. For example, wild boars from Jeolla-do, in the southwest region of South Korea, shared only 3.6% genetic composition with the population from Gyeonggi-do in the northwest. This result indicates that wild boar distribution and partial isolation in the Korean peninsula are possibly maintained by geographic barriers such as mountain ridges, lowlands and islands. Although wild boars are now abundant in South Korea, various levels of genetic and ecological studies will be required to obtain adequate information for long-term management.

## Conclusions

Microsatellite loci analyses revealed wild boar populations of East Asia are genetically diverse and structured, and that genetic distance is correlated with geographic distance. The level of genetic diversity decreases gradually from Southeastern Asia to Northeastern Asia, reflecting northward spread of ancestral wild boar populations in East Asia. We also observed conspicuous genetic structure and divergence among wild boar populations at local and regional scales in East Asia. High levels and diverse patterns of genetic variation among regional populations of wild boars from East Asia have likely contributed to the high genetic diversity of local domestic pig populations retained through multiple independent domestications [3]. In addition, extant genetic richness of wild boars in East Asia can become an important resource for the future breeding of domestic pigs. Although microsatellites provide genetic information other markers do not, novel approaches such as SNP and genome sequencing also will be helpful in better understanding the population structure of wild boars in East Asia. Moreover, further studies with more samples at larger and finer geographic scales will shed light on unresolved questions, such as the paternal and maternal history, and the phylogeography of wild boars from Eurasia. Such studies are currently underway.

## Methods

### Sample collection

Samples from a total of 238 wild boars, mostly muscle tissue, some blood and hair, were collected from ten locations across six countries; Russia (Primorsky Krai, RUPW), Japan (JPNW), China (Yunnan province, CYNW), Vietnam (VIEW), Indonesia (INDW) and South Korea. This experimental work was conducted with permission by the Conservation Genome Resource Bank for Korean Wildlife (CGRB) that provided wild boar samples for this study. All samples were legally collected and deposited into CGRB. The procedures involving animal samples followed the guidelines by Seoul National University Institutional Animal Care and Use Committee (SNUIACUC). Wild boars in South Korea were divided into five regional groups according to the province of collection and other geographic considerations: Gyeonggi-do (KGGW), Gangwon-do (KGWW), Gyeongsang-do (KGSW), Jeolla-do (KJLW) and Jeju Island (KJIW) (Table 1, Figure 2). All the samples were stored at −70°C until DNA extraction.

### Microsatellite markers and PCR

In this study, we selected and tested 18 of 30 polymorphic microsatellite markers developed for swine biodiversity studies [28]. We carried out a series of tests using a subset of Korean wild boars to verify if these markers adequately fit marker selection criteria suggested by Kim *et al.*[29]. 16 of 18 markers revealed good scorability, Hardy–Weinberg equilibrium, absence of null alleles, evidence of selective neutrality and linkage equilibrium between loci. Therefore, these 16 markers were used for wild boar population genetics in this study. Information on genetic variation for individual markers from wild boars sampled at each location is shown in (Additional file 1: Table S1).

Genomic DNA was extracted using the DNeasy Blood & Tissue Kit or Gentra Puregene Tissue Kit (QIAGEN) according the manufacturer’s instructions. The 16 microsatellite loci were amplified using the Multiplex PCR Kit (QIAGEN). Touchdown PCR was carried out under the following conditions: initial denaturation for 15 min at 95°C, followed by seven touchdown cycles starting at 94°C for 30s, 67°C for 90s, and 72°C for 60s, with annealing temperature decreasing by 2°C per cycle to 53°C. The touchdown cycles were followed by an additional 25 cycles at 94°C for 30s, 53°C for 90s, 72°C for 60s, and a final extension at 60°C for 30 min. Individuals were genotyped using a DNA Sequencer (ABI Prism 3730 XL DNA Analyzer, Applied Biosystems).

### Data analysis

Measures of genetic diversity, including mean number of alleles (*Ad*) per locus, observed heterozygosity (*H*_O_), and expected heterozygosity (*H*_E_) under Hardy-Weinberg assumptions, were estimated using the Microsatellite Tool Kit [30]. Allelic richness (*Ar*) [31] is a fundamental measure of genetic diversity. It was calculated based on the minimum sample size of each population to correct for differences in sample size among populations using the rarefaction approach implemented in FSTAT v. 2.9.3 [32]. Inbreeding coefficient, *F*_IS_, and the level of genetic differentiation between each pair of populations, pairwise *F*_ST_ estimates, and their significance values were calculated using a permutation approach with FSTAT v. 2.9.3 [32]. Significance level was determined after applying the sequential Bonferroni correction to take account of experiment-wise errors due to multiple tests [33]. Indirect estimates of gene flow (*Nm*, effective number of migrants per generation) were calculated from *F*_ST_ using the equation of Wright [34]. The software program GenAlEx v.6.0 [35] was used to conduct Principal Coordinates Analysis (PCA) to visualize geometric relationships between wild boar populations. GenAlEx v.6.0 was further used to carry out an analysis of molecular variance (AMOVA) for wild boars among three potential regions suggested by the model-based clustering analysis (see Results): North-East (RUPW, KGGW, KGWW, KGSW, KJLW and JPNW), Jeju Island (KJIW) and South-East (CYNW, VIEW and INDW). Significance level was calculated by the permutation procedure (999 permutations). We checked for isolation by distance (IBD) [34] by testing for correlation between genetic distance, *F*_ST_/(1-*F*_ST_), and geographic distance among locations using Mantel’s test in GenAlEx v.6.0, and significance was determined based on 999 permutations. The DISPAN computer program [36] was used to construct the genetic relationship tree based on Nei’s D_A_ genetic distance by the neighbor-joining (NJ) method [37,38].

To assess population structure, STRUCTURE 2.3.3 software [39] was used. The number of MCMC (Markov chain Monte Carlo) replications was set to 200,000 after a burn-in period of 100,000 using the default parameters of an admixture model and correlated allele frequencies among populations. The number of inferred clusters (*K*) was estimated according to the method of Evanno *et al*. [13], where an ad hoc statistic Δ*K* is based on the rate of change in the log probability of data between successive *K* values. Ten runs were carried out for each *K*, from 1 to 12, to quantify the amount of variation of the likelihood value. Initially, we obtained the highest Δ*K* value when *K* was set to 3 (see Results). Three main clusters, therefore, were further analyzed according to the hierarchical island model to probe for possible hidden substructure for each predefined cluster [13].

## Competing interests

The authors declare that they have no competing interests.

## Authors’ contributions

SKC carried out the molecular genetic studies, participated in the experiments, data analyses, and drafted the manuscript. KSK and HL conceived of the study, and participated in its design and coordination and helped to draft the manuscript. JEL participated in the experiment. YJK, MSM, IV, AM, JGO, THK, IS, NI, LY, YPZ and NM provided genetic materials and helped to draft the manuscript. All authors read and approved the final manuscript.

## Supplementary Material

Additional file 1: Figure S1 Plot of mean posterior probability (LnP(D)) values per clusters (*K*), based on 10 iterations per *K*, generated by the STRUCTURE program [39], and delta *K* analysis of LnP(D), according to Evanno *et al.*[13]. **Figure S2**: Regression of genetic distance on geographic distance between pairs of East Asian wild boar populations. A. Analysis using all populations included (*P* = 0.140); B. Analysis after excluding wild boars from Jeju Island (*P* = 0.002). Mantel’s test for correlations was carried out with 999 permutations. **Table S1**: Genetic characteristics of 16 microsatellite DNA loci for ten sampling locations in East Asia. See Table 1 for sample locations. Click here for file
