# Supplementary material for: Genetic structure of wild boar (Sus scrofa) populations from East Asia based on microsatellite loci analyses
Source: BMC Genet. 2014 Jul 17;15:85. doi: 10.1186/1471-2156-15-85 (PMC4112609; doi:10.1186/1471-2156-15-85)
Supplement: Additional file 1: Figure S1 — Plot of mean posterior probability (LnP(D)) values per clusters (K), based on 10 iterations per K, generated by the STRUCTURE program [39], and delta K analysis of LnP(D), according to Evanno et al.[13]. Figure S2: Regression of genetic distance on geographic distance between pairs of East Asian wild boar populations. A. Analysis using all populations included (P = 0.140); B. Analysis after excluding wild boars from Jeju Island (P = 0.002). Mantel’s test for correlations was carried out with 999 permutations. Table S1: Genetic characteristics of 16 microsatellite DNA loci for ten sampling locations in East Asia. See Table 1 for sample locations. [file 1471-2156-15-85-S1.doc]

**SUPPORTING INFORMATION**

**Genetic Structure of Wild Boar (*Sus scrofa*) Populations from East Asia Based on Microsatellite Loci Analyses**

Sung Kyoung Choi, Ji-Eun Lee, Young-Jun Kim, Mi-Sook Min, Inna Voloshina, Alexander Myslenkov, Jang Geun Oh, Tae-Hun Kim, Nickolay Markov, Ivan Seryodkin, Naotaka Ishiguro, Li Yu, Ya-Ping Zhang, Hang Lee^*^ and Kyung Seok Kim^*^

**Figure S1.** Plot of mean posterior probability (LnP(D)) values per clusters (*K*), based on 10 iterations per *K*, generated by the STRUCTURE program [41], and delta *K* analysis of LnP(D), according to Evanno *et al.*, (2005)[13].


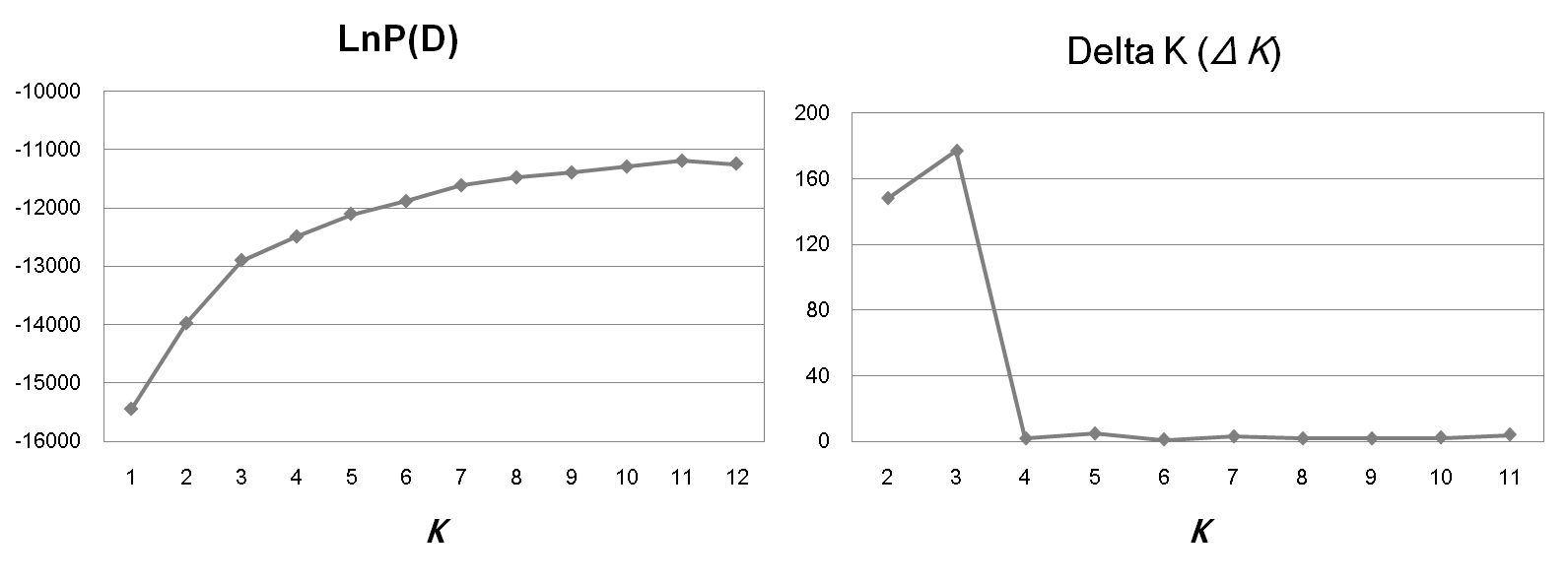


**Figure S2.** Regression of genetic distance on geographic distance between pairs of East Asian wild boar populations. A. Analysis using all populations included (*P*=0.140); B. Analysis using wild boars from Jeju Island excluded (*P*=0.002). Mantel’s test for correlations was carried out with 999 permutations.


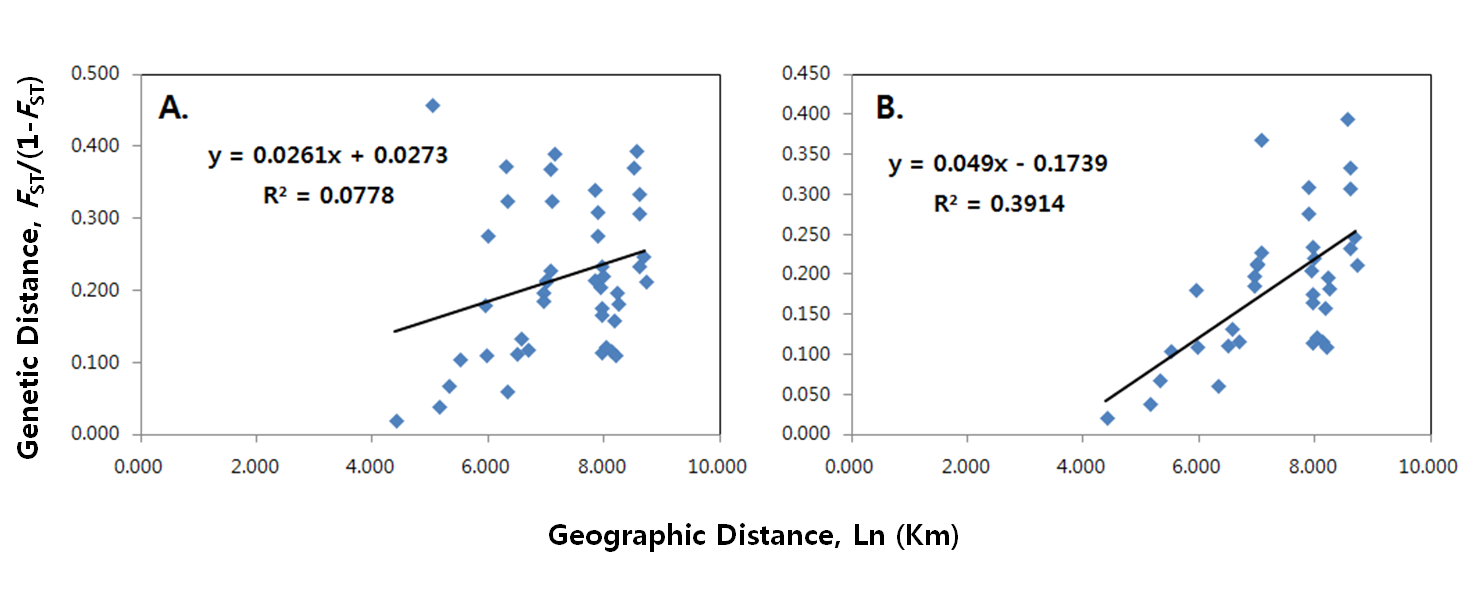


**Table S1**. Genetic characteristics of 16 microsatellite DNA loci for ten sampling locations in East Asia. See Table 1 for sample locations.

| Locus | KGGW  (n=17) | KGWW (n=53) | KGSW (n=26) | KJLW (n=12) | KJIW (n=37) | RUPW (n=30) | JPNW (n=16) | CYNW (n=10) | VIEW (n=13) | INDW (n=24) | Mean |
| --- | --- | --- | --- | --- | --- | --- | --- | --- | --- | --- | --- |
| **S0026** |  |  |  |  |  |  |  |  |  |  |  |
| No. of allele | 7 | 6 | 8 | 4 | 4 | 10 | 7 | 6 | 10 | 8 | 7 |
| *H*_O_ | 0.529 | 0.811 | 0.923 | 0.583 | 0.595 | 0.700 | 0.375 | 0.800 | 0.923 | 0.667 | 0.691 |
| *H*_E_ | 0.697 | 0.800 | 0.845 | 0.678 | 0.655 | 0.867 | 0.599 | 0.847 | 0.880 | 0.780 | 0.765 |
| HWE P-value | 0.0842 | 0.0198 | 0.1677 | 0.0137 | 0.3832 | 0.0204 | 0.0074 | 0.7204 | 0.0871 | 0.0155 | - |
| **S0155** |  |  |  |  |  |  |  |  |  |  |  |
| No. of allele | 4 | 6 | 5 | 3 | 4 | 7 | 7 | 7 | 10 | 5 | 5.8 |
| *H*_O_ | 0.647 | 0.660 | 0.577 | 0.417 | 0.703 | 0.633 | 0.625 | 0.800 | 0.923 | 0.375 | 0.636 |
| *H*_E_ | 0.704 | 0.665 | 0.637 | 0.627 | 0.635 | 0.705 | 0.841 | 0.853 | 0.806 | 0.527 | 0.700 |
| HWE P-value | 0.1179 | 0.0189 | 0.5719 | 0.2154 | 0.0635 | 0.2549 | 0.2365 | 0.2744 | 0.5148 | 0.0006 | - |
| **S0005** |  |  |  |  |  |  |  |  |  |  |  |
| No. of allele | 2 | 4 | 10 | 1 | 4 | 9 | 6 | 15 | 12 | 17 | 8 |
| *H*_O_ | 0.176 | 0.377 | 0.500 | 0.000 | 0.622 | 0.600 | 0.563 | 1.000 | 0.692 | 0.708 | 0.524 |
| *H*_E_ | 0.166 | 0.369 | 0.666 | 0.000 | 0.704 | 0.710 | 0.641 | 0.968 | 0.895 | 0.778 | 0.590 |
| HWE P-value | 1.0000 | 0.3987 | 0.0081 | NA | 0.0012 | 0.0400 | 0.0046 | 1.0000 | 0.0000 | 0.0113 | - |
| **Sw24** |  |  |  |  |  |  |  |  |  |  |  |
| No. of allele | 6 | 6 | 8 | 5 | 3 | 9 | 7 | 10 | 12 | 8 | 7.4 |
| *H*_O_ | 0.765 | 0.811 | 0.962 | 0.583 | 0.432 | 0.733 | 0.688 | 0.800 | 0.846 | 0.708 | 0.733 |
| *H*_E_ | 0.799 | 0.784 | 0.834 | 0.768 | 0.450 | 0.779 | 0.778 | 0.879 | 0.926 | 0.791 | 0.779 |
| HWE P-value | 0.2338 | 0.8868 | 0.1949 | 0.4073 | 0.8451 | 0.1062 | 0.3349 | 0.6881 | 0.0097 | 0.0170 | - |
| **Sw632** |  |  |  |  |  |  |  |  |  |  |  |
| No. of allele | 4 | 7 | 6 | 3 | 3 | 11 | 6 | 6 | 7 | 9 | 6.2 |
| *H*_O_ | 0.412 | 0.528 | 0.808 | 0.250 | 0.459 | 0.667 | 0.188 | 1.000 | 0.692 | 0.458 | 0.546 |
| *H*_E_ | 0.579 | 0.465 | 0.713 | 0.522 | 0.530 | 0.734 | 0.581 | 0.779 | 0.769 | 0.670 | 0.634 |
| HWE P-value | 0.0070 | 0.1419 | 0.3042 | 0.0524 | 0.0182 | 0.0760 | 0.0000 | 0.0553 | 0.1196 | 0.0000 | - |
| **Swr1941** |  |  |  |  |  |  |  |  |  |  |  |
| No. of allele | 3 | 4 | 4 | 2 | 3 | 3 | 6 | 8 | 7 | 7 | 4.7 |
| *H*_O_ | 0.235 | 0.528 | 0.615 | 0.000 | 0.784 | 0.300 | 0.500 | 0.900 | 0.923 | 0.750 | 0.554 |
| *H*_E_ | 0.392 | 0.585 | 0.646 | 0.159 | 0.643 | 0.402 | 0.706 | 0.884 | 0.800 | 0.787 | 0.600 |
| HWE P-value | 0.1010 | 0.0793 | 0.2756 | 0.0435 | 0.0709 | 0.1304 | 0.0282 | 0.9626 | 0.3875 | 0.1704 | - |
| **Sw122** |  |  |  |  |  |  |  |  |  |  |  |
| No. of allele | 5 | 5 | 6 | 3 | 6 | 6 | 5 | 9 | 9 | 11 | 6.5 |
| *H*_O_ | 0.529 | 0.642 | 0.692 | 0.417 | 0.595 | 0.667 | 0.313 | 1.000 | 1.000 | 0.875 | 0.673 |
| *H*_E_ | 0.740 | 0.630 | 0.733 | 0.540 | 0.710 | 0.658 | 0.587 | 0.916 | 0.852 | 0.902 | 0.727 |
| HWE P-value | 0.0612 | 0.9020 | 0.0813 | 0.3970 | 0.0068 | 0.1188 | 0.0013 | 0.7953 | 0.2588 | 0.0000 | - |
| **Sw857** |  |  |  |  |  |  |  |  |  |  |  |
| No. of allele | 4 | 4 | 4 | 2 | 5 | 6 | 4 | 8 | 12 | 13 | 6.2 |
| *H*_O_ | 0.529 | 0.698 | 0.577 | 0.083 | 0.595 | 0.867 | 0.625 | 0.900 | 0.923 | 0.458 | 0.626 |
| *H*_E_ | 0.683 | 0.668 | 0.562 | 0.083 | 0.674 | 0.720 | 0.623 | 0.889 | 0.923 | 0.885 | 0.671 |
| HWE P-value | 0.3617 | 0.0005 | 0.0640 | NA | 0.0588 | 0.5226 | 0.2214 | 0.1138 | 0.0048 | 0.0000 | - |
| **sw240** |  |  |  |  |  |  |  |  |  |  |  |
| No. of allele | 6 | 6 | 4 | 3 | 3 | 9 | 11 | 9 | 10 | 12 | 7.3 |
| *H*_O_ | 0.882 | 0.792 | 0.615 | 0.417 | 0.027 | 0.800 | 0.688 | 0.800 | 0.769 | 0.792 | 0.658 |
| *H*_E_ | 0.806 | 0.827 | 0.687 | 0.475 | 0.054 | 0.827 | 0.867 | 0.911 | 0.911 | 0.859 | 0.722 |
| HWE P-value | 0.7601 | 0.0693 | 0.4072 | 0.2677 | 0.0137 | 0.2287 | 0.0179 | 0.5300 | 0.0006 | 0.0224 | - |
| **IGF1** |  |  |  |  |  |  |  |  |  |  |  |
| No. of allele | 5 | 6 | 5 | 4 | 3 | 8 | 7 | 7 | 6 | 8 | 5.9 |
| *H*_O_ | 0.706 | 0.547 | 0.462 | 0.583 | 0.541 | 0.667 | 0.500 | 0.800 | 0.769 | 0.833 | 0.641 |
| *H*_E_ | 0.554 | 0.498 | 0.540 | 0.576 | 0.582 | 0.737 | 0.734 | 0.832 | 0.806 | 0.866 | 0.673 |
| HWE P-value | 0.7652 | 0.8540 | 0.1475 | 0.6769 | 0.4725 | 0.5390 | 0.0018 | 0.7547 | 0.1052 | 0.0005 | - |
| **Sw72** |  |  |  |  |  |  |  |  |  |  |  |
| No. of allele | 5 | 5 | 4 | 4 | 3 | 6 | 4 | 6 | 7 | 6 | 5 |
| *H*_O_ | 0.647 | 0.698 | 0.731 | 0.500 | 0.676 | 0.733 | 0.500 | 0.700 | 0.538 | 0.583 | 0.631 |
| *H*_E_ | 0.709 | 0.752 | 0.746 | 0.540 | 0.519 | 0.716 | 0.659 | 0.832 | 0.862 | 0.812 | 0.715 |
| HWE P-value | 0.0436 | 0.2678 | 0.4934 | 0.2591 | 0.0663 | 0.5088 | 0.2447 | 0.8422 | 0.0039 | 0.0075 | - |
| **S0226** |  |  |  |  |  |  |  |  |  |  |  |
| No. of allele | 5 | 6 | 6 | 4 | 6 | 11 | 5 | 8 | 12 | 9 | 7.2 |
| *H*_O_ | 0.765 | 0.660 | 0.462 | 0.500 | 0.730 | 0.833 | 0.313 | 0.600 | 0.923 | 0.750 | 0.654 |
| *H*_E_ | 0.729 | 0.732 | 0.592 | 0.424 | 0.683 | 0.902 | 0.613 | 0.784 | 0.914 | 0.810 | 0.718 |
| HWE P-value | 0.4868 | 0.1650 | 0.0334 | 1.0000 | 0.0502 | 0.0829 | 0.0101 | 0.3792 | 0.0107 | 0.0027 | - |
| **S0090** |  |  |  |  |  |  |  |  |  |  |  |
| No. of allele | 6 | 6 | 7 | 6 | 3 | 7 | 4 | 6 | 8 | 11 | 6.4 |
| *H*_O_ | 0.765 | 0.698 | 0.654 | 0.917 | 0.000 | 0.900 | 0.188 | 0.700 | 0.769 | 0.833 | 0.642 |
| *H*_E_ | 0.622 | 0.652 | 0.801 | 0.841 | 0.105 | 0.844 | 0.181 | 0.684 | 0.868 | 0.848 | 0.645 |
| HWE P-value | 0.3775 | 0.5284 | 0.1221 | 0.4948 | 0.0002 | 0.4145 | 1.0000 | 0.7436 | 0.0250 | 0.0000 | - |
| **Sw1828** |  |  |  |  |  |  |  |  |  |  |  |
| No. of allele | 4 | 6 | 5 | 4 | 5 | 7 | 8 | 5 | 7 | 8 | 5.9 |
| *H*_O_ | 0.353 | 0.635 | 0.692 | 0.583 | 0.568 | 0.767 | 0.250 | 0.600 | 0.917 | 0.522 | 0.589 |
| *H*_E_ | 0.528 | 0.654 | 0.763 | 0.710 | 0.644 | 0.673 | 0.637 | 0.753 | 0.768 | 0.835 | 0.697 |
| HWE P-value | 0.1958 | 0.0178 | 0.0362 | 0.3961 | 0.0080 | 0.1488 | 0.0000 | 0.3232 | 0.2660 | 0.0000 | - |
| **S0143** |  |  |  |  |  |  |  |  |  |  |  |
| No. of allele | 5 | 7 | 7 | 3 | 5 | 6 | 4 | 6 | 8 | 10 | 6.1 |
| *H*_O_ | 0.412 | 0.623 | 0.692 | 0.500 | 0.622 | 0.667 | 0.563 | 0.900 | 0.769 | 0.708 | 0.646 |
| *H*_E_ | 0.480 | 0.739 | 0.738 | 0.681 | 0.585 | 0.710 | 0.603 | 0.758 | 0.855 | 0.848 | 0.700 |
| HWE P-value | 0.3824 | 0.0001 | 0.0704 | 0.1001 | 0.6810 | 0.0851 | 0.5620 | 0.8287 | 0.0367 | 0.0000 | - |
| **S0068** |  |  |  |  |  |  |  |  |  |  |  |
| No. of allele | 6 | 8 | 8 | 4 | 4 | 7 | 8 | 12 | 9 | 12 | 7.8 |
| *H*_O_ | 0.647 | 0.642 | 0.808 | 0.417 | 0.676 | 0.833 | 0.688 | 1.000 | 1.000 | 0.500 | 0.721 |
| *H*_E_ | 0.638 | 0.760 | 0.770 | 0.471 | 0.612 | 0.794 | 0.746 | 0.947 | 0.908 | 0.735 | 0.738 |
| HWE P-value | 0.5612 | 0.0003 | 0.3907 | 0.7071 | 0.0098 | 0.0214 | 0.4620 | 1.0000 | 0.2153 | 0.0000 | - |

n: Sample size; *H*_O_: Observed heterozygosity; *H*_E_: Expected heterozygosity; HWE: Hardy-Weinberg equilibrium; NA: Not available.
